# Multi-strategy endoscopic submucosal dissection of a challenging lesion

**DOI:** 10.1055/a-2517-1878

**Published:** 2025-02-06

**Authors:** Véronique Van der Voort, Romain Legros, Jérémie Albouys, Hugo Lepetit, Timothée Wallenhorst, Mathieu Pioche, Jérémie Jacques

**Affiliations:** 1 36715 Department of Gastroenterology and Hepatology, Centre Hospitalier Universitaire de Limoges, Limoges, France; 2 Department of Gastroenterology and Hepatology, Centre Hospitalier Universitaire de Rennes, Site de Pontchaillou, Rennes, France; 3 36609 Department of Gastroenterology and Hepatology, Hôpital Edouard Herriot, Lyon, France

Over the years, advancements in endoscopic submucosal dissection (ESD) have significantly refined and simplified the technique, allowing for successful endoscopic resection of increasingly complex lesions. Determining the right strategy or tool can be challenging and is not bound by strict rules – sometimes, in complex cases, you end up using every tool available.

A 79-year-old woman was referred for endoscopic resection of a 25-mm, Paris-IIa non-granular lesion, described as a sessile serrated lesion. Assessment with virtual chromoendoscopy revealed a focal JNET-2B pattern, raising suspicion of superficial submucosal invasive cancer, leading to the decision to perform ESD ( Video 1 ). The lesion was situated in a challenging position in the right colon, where scope maneuverability was poor. The saline-immersion technique was utilized to stabilize the scope and improve lesion assessment 1 . Circumferential incision and trimming were performed with a 1.5-mm HYBRIDknife flex T-type (Erbe Elektromedizin Gmbh, Tübingen, Germany), while also benefiting from the buoyancy effect of the saline 2 . A double-clip and rubber band were placed (while still underwater) to create traction and improve exposure of the plane ( Fig. 1 **a, b** ) 3 . High-pressure injection through the knife allowed efficient submucosal lifting, and dissection was performed using EndoCut I mode (VIO 3, Erbe Elektromedizin GmbH). Large vessels were precoagulated with the plate at the tip of the knife using SoftCoag mode ( Fig. 1 **d** ). Traction with the tip of the knife of the submucosal fibers allowed safe completion of the dissection ( Fig. 1 **c** ). The 50 × 30-mm specimen was successfully removed en bloc in 28 minutes.

Endoscopic submucosal dissection of a suspicious lesion in the right colon in a difficult position, involving multiple strategies and tools to remove the lesion en bloc.Video 1

**Fig. 1:**
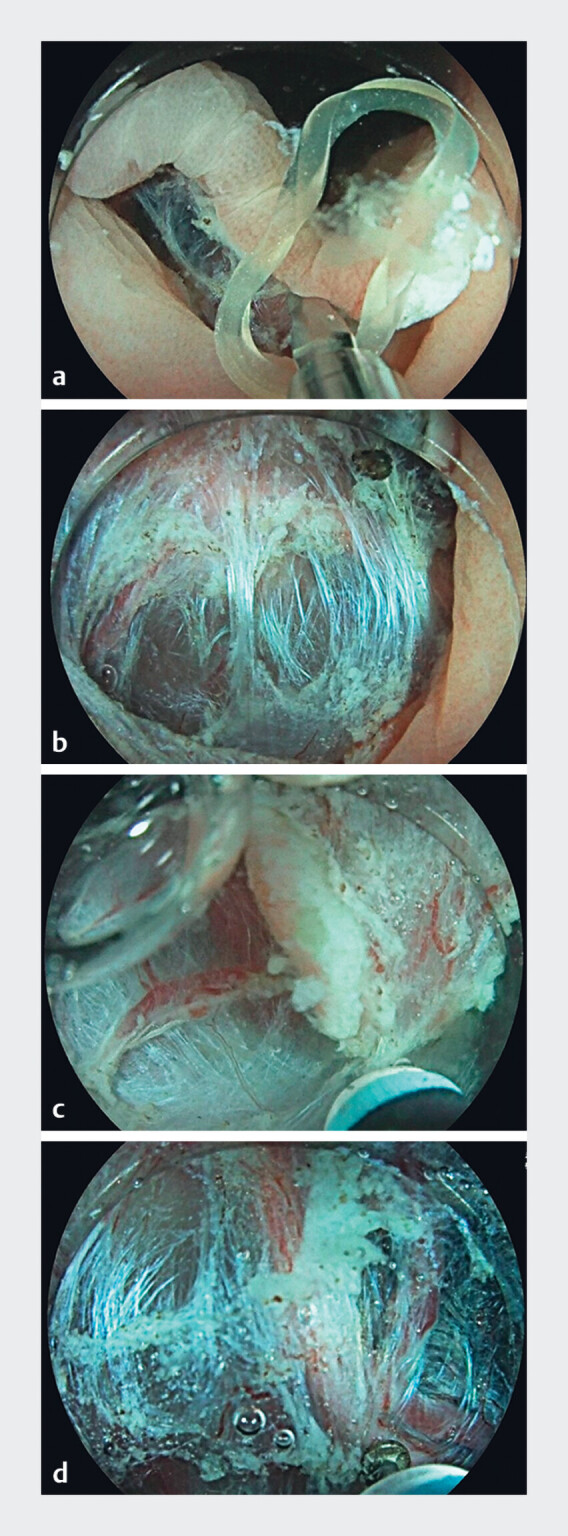
**a** After completing the circumferential incision, a clip and rubber band are placed at the edge of the lesion to provide traction in the submucosal plane. **b** Traction is created (while still underwater) on the exposed submucosal fibers, making the dissection plane clearly visible. **c** The plate on the tip of the knife is used to hook, apply traction to, and cut the submucosal fibers in a precise way. **d** Large vessels are preemptively coagulated with the plate on the tip of the knife before cutting.

Histology revealed R0-resection of a poorly differentiated pT1 adenocarcinoma with 930-µm submucosal invasion, presence of lymphatic invasion and low grade tumor budding ( Fig. 2 ). Absence of local or distant metastasis was confirmed by computed tomography, and the patient was referred for completion surgery.

**Fig. 2:**
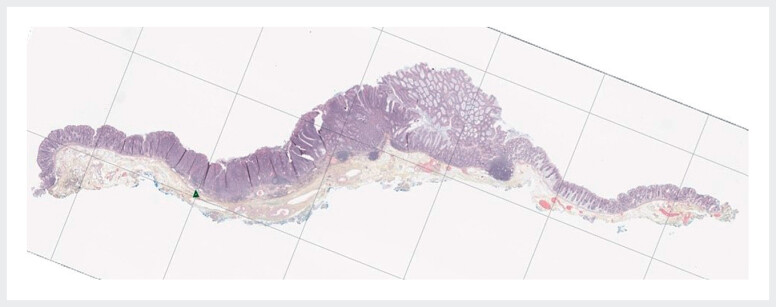
Histological image of the pT1 adenocarcinoma of the right colon, with presence of high risk features.

Although the different strategies used to optimize ESD are often presented as competitors, we believe that they should be combined because of their complementary advantages.

Endoscopy_UCTN_Code_TTT_1AQ_2AD_3AD

## Notes

Endoscopy E-Videos https://eref.thieme.de/e-videos
*E-Videos* is an open access online section of the journal *Endoscopy* , reporting on interesting cases and new techniques in gastroenterological endoscopy. All papers include a high-quality video and are published with a Creative Commons CC-BY license. Endoscopy E-Videos qualify for HINARI discounts and waivers and eligibility is automatically checked during the submission process. We grant 100% waivers to articles whose corresponding authors are based in Group A countries and 50% waivers to those who are based in Group B countries as classified by Research4Life (see: https://www.research4life.org/access/eligibility/ ). This section has its own submission website at https://mc.manuscriptcentral.com/e-videos .

## References

[1] Lambin T Rivory J Wallenhorst T Endoscopic submucosal dissection: How to be more efficient? Endosc Int Open 2021 9 E1720 E1730 10.1055/a-1554-3884 34790536 PMC8589544

[2] Nagata M Usefulness of underwater endoscopic submucosal dissection in saline solution with a monopolar knife for colorectal tumors Gastrointest Endosc 2018 87 1345 1353 10.1016/j.gie.2017.11.032 29242059

[3] Jacques J Charissoux A Bordillon P High proficiency of colonic endoscopic submucosal dissection in Europe thanks to countertraction strategy using a double clip and rubber band Endosc Int Open 2019 7 E1166 E1174 10.1055/a-0965-8531 31475236 PMC6715438

